# Bloody nipple discharge in young children: mammary duct ectasia as a benign, self-limiting condition

**DOI:** 10.1093/jscr/rjag028

**Published:** 2026-01-30

**Authors:** Mohammed Al Blooshi, Dalia D Abdulrahman, Wisam Salih Saad, Usman Javaid, Vipul Gupta

**Affiliations:** Department of Pediatric Surgery, Al Jalilah Hospital, Dubai, United Arab Emirates; Dubai Medical University, Dubai, United Arab Emirates; Clinical Attendee, Pediatric Surgery, Al Jalila Children’s Specialty Hospital, Dubai, United Arab Emirates; Department of Pediatric Surgery and Urology, Al Jalila Children’s Specialty Hospital, Dubai, United Arab Emirates; Department of Pediatric Surgery and Urology, Al Jalila Children’s Specialty Hospital, Dubai, United Arab Emirates

**Keywords:** bloody nipple discharge, pediatrics, mammary duct ectasia, breast ultrasonography, conservative management

## Abstract

Bloody nipple discharge in children is rare and often alarms families because of its malignant associations in adults. We describe two prepubertal boys (3 and 5 years) with unilateral, spontaneous, painless bloody nipple discharge without trauma, infection, systemic symptoms, or exogenous hormone exposure. Examinations showed only mild unilateral breast change, with no masses, skin or nipple abnormalities, or lymphadenopathy. Breast ultrasonography was the primary investigation: one child had retroareolar ductal dilatation with cystic change consistent with mammary duct ectasia; the other had normal breast architecture. When obtained, laboratory, microbiologic, and cytologic studies were unremarkable. Both patients were managed conservatively with caregiver counseling and structured clinical/sonographic follow-up. Discharge resolved spontaneously within 14 and 8 months, respectively, with complete clinical and imaging resolution and no recurrence. These cases support ultrasound-based assessment and expectant management when no concerning features are present.

## Introduction

Breast disorders are uncommon in the pediatric population, and when present, they are most frequently benign and self-limiting. The most commonly encountered nipple discharge in infants and young children is milky secretion, typically observed during the neonatal period and attributed to transient hormonal influences related to maternal estrogen exposure and postnatal endocrine adaptation. This phenomenon is generally well recognized by clinicians and caregivers and rarely prompts extensive investigation or intervention.

In contrast, bloody nipple discharge in children is an exceedingly rare clinical presentation and often generates significant concern among caregivers and healthcare providers alike. In adult patients, bloody nipple discharge is classically associated with underlying breast pathology, including intraductal papilloma and malignancy, which contributes to heightened anxiety when the same symptom occurs in a child. As a result, children presenting with bloody nipple discharge are at risk of undergoing unnecessary diagnostic investigations or invasive procedures despite the markedly different etiological spectrum in the pediatric age group [1–4].

Available literature consistently demonstrates that bloody nipple discharge in children is most often a benign condition with a favorable natural course. The majority of reported cases occur in infants and young children, with a notable male predominance, and typically present as unilateral, painless, intermittent discharge without associated systemic symptoms or signs of local inflammation [1, 5–7]. Mammary duct ectasia has emerged as the most frequently identified underlying abnormality and is characterized by ductal dilatation with periductal inflammation or fibrosis. Importantly, this condition differs fundamentally from adult duct ectasia in both pathophysiology and clinical implications and is not associated with malignant transformation in children [1, 8].

Ultrasonography plays a pivotal role in the evaluation of pediatric patients with bloody nipple discharge. Owing to its noninvasive nature and excellent soft tissue resolution, breast ultrasound is considered the imaging modality of choice and has largely replaced surgical exploration as the primary diagnostic approach. Typical sonographic findings include retroareolar ductal dilatation and cystic changes without suspicious solid components or vascularity, allowing confident diagnosis and reassurance of caregivers [4, 6–9]. With increasing awareness of these characteristic imaging features, conservative management strategies emphasizing observation and parental counseling have become widely accepted.

Despite the benign nature of this condition, a lack of familiarity among primary care physicians and pediatric specialists may still lead to over-investigation or premature surgical intervention. Surgical excision, when performed, has not demonstrated additional clinical benefit and carries the potential risk of disrupting normal breast development. Contemporary evidence supports expectant management with serial clinical and sonographic follow-up, as spontaneous resolution typically occurs within months [1, 5, 7, 8].

In this report, we present two cases of bloody nipple discharge in young male children, highlighting their clinical presentation, imaging findings, management strategy, and outcomes. Through these cases, we aim to reinforce current understanding of this rare but benign entity and emphasize the importance of conservative management and parental reassurance in pediatric patients presenting with bloody nipple discharge.

## Case series

### Case 1

A previously healthy 3-year-old boy was brought to the pediatric surgery clinic with a history of intermittent bloody discharge from the left nipple. The discharge was first noticed by the parents several days prior to presentation and was spontaneous in onset, occurring without nipple manipulation or preceding trauma. There was no associated fever, pain, erythema, or systemic symptoms. The child had no history of medication use or exposure to hormonal preparations. Family history was notable for breast carcinoma in a maternal aunt, which significantly heightened parental concern.

On clinical examination, the child was well and developmentally appropriate for age. Local examination of the left breast revealed mild unilateral breast enlargement with a small, ill-defined, retroareolar nodularity. The area was non-tender, and the overlying skin and nipple appeared normal without retraction, discoloration, or ulceration. Gentle inspection revealed traces of blood-stained discharge at the nipple. No axillary lymphadenopathy was detected. Examination of the contralateral breast was unremarkable.

Following initial assessment, a structured diagnostic evaluation was undertaken to exclude inflammatory, infectious, hormonal, or malignant etiologies. Laboratory investigations, including complete blood count and inflammatory markers, were within normal limits. Endocrine evaluation comprising estradiol, progesterone, and prolactin levels demonstrated no abnormalities. Microbiological analysis of the nipple discharge revealed no pathogenic organisms, and cytological examination showed no evidence of malignant or inflammatory cells.

**Figure 1:**
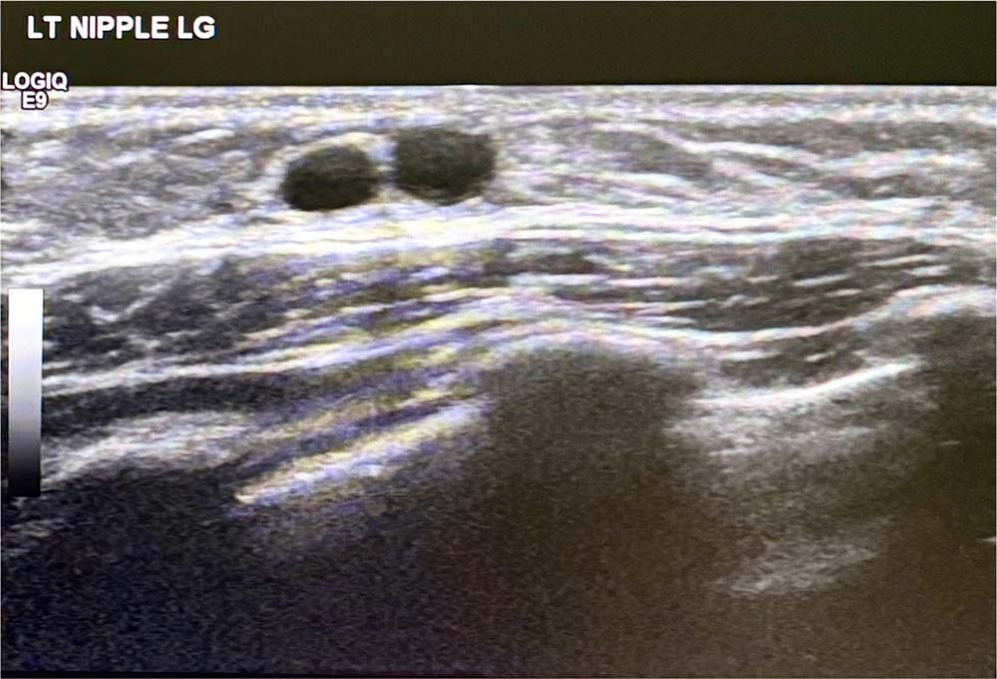
Ultrasound scan left breast showing cystic duct changes suggestive of mammary duct ectasia.

Targeted ultrasonography of the left breast was subsequently performed as the primary imaging modality. Sonographic evaluation demonstrated a small hypoechoic retroareolar lesion measuring ~10 by 2 millimeters with internal cystic ductal changes and absence of internal vascularity on color Doppler assessment. These findings were consistent with mammary duct ectasia and are illustrated in Fig. 1.

In view of the benign clinical and radiological findings, a conservative management strategy was adopted. The parents were extensively counseled regarding the benign and self-limiting nature of the condition, and the child was enrolled in a structured follow-up program with periodic clinical assessments and serial breast ultrasonography.

Over subsequent follow-up visits, a gradual reduction in both the frequency of nipple discharge and the size of the palpable retroareolar nodularity was observed. Fourteen months after the initial presentation, the bloody discharge had completely resolved. Follow-up clinical examination and ultrasonographic assessment performed six months later confirmed complete resolution of the lesion with restoration of normal breast architecture. The child remained asymptomatic with no recurrence at final review.

### Case 2

A 5-year-old boy presented with a 2-month history of intermittent blood-stained nipple discharge from the right breast. The discharge was noted to be spontaneous and painless, with no associated swelling, redness, or local discomfort. There was no history of trauma, fever, systemic illness, or medication exposure. Unlike the first case, there was no family history of breast malignancy.

Physical examination revealed a well-appearing child with normal vital parameters. Inspection of the right breast demonstrated minimal blood-tinged serous discharge from the nipple without visible skin or nipple abnormalities. No palpable mass was identified, and there was no tenderness or lymphadenopathy. Examination of the left breast was normal.

Given the benign clinical appearance and prior institutional experience with a similar presentation, emphasis was placed on parental reassurance and observation. Ultrasonographic evaluation of the right breast was performed to exclude underlying structural pathology and demonstrated normal breast tissue architecture with no focal lesion or ductal abnormality.

The child was managed expectantly with close outpatient follow-up. The parents received detailed counseling regarding the anticipated clinical course and the absence of features suggestive of malignancy or infection. No laboratory or hormonal investigations were pursued.

During follow-up, the frequency of discharge progressively diminished and resolved completely within eight months of initial presentation. Serial clinical assessments and sonographic evaluations remained normal throughout the observation period. The child continued to be asymptomatic at one-year follow-up, with no evidence of recurrence.

## Discussion

Bloody nipple discharge in children represents a rare but clinically striking presentation that often prompts disproportionate concern due to its well-established association with malignant and premalignant breast conditions in adults. In contrast, pediatric cases demonstrate a fundamentally different clinical behavior, etiology, and prognosis. The present report reinforces existing evidence that bloody nipple discharge in young children is a benign, self-limiting condition, most commonly related to mammary duct ectasia, and can be safely managed with conservative measures and structured follow-up.

Both cases in this series illustrate the typical clinical pattern described in pediatric literature. The presentation was characterized by spontaneous, painless, unilateral bloody discharge occurring in young male children without associated inflammatory signs, systemic symptoms, or significant endocrine abnormalities. This constellation of findings aligns with prior observations that pediatric bloody nipple discharge predominantly affects boys, often before the age of 5, and follows a benign clinical course [1, 5, 7, 8]. The male predominance remains unexplained, though developmental differences in ductal anatomy and hormonal responsiveness have been postulated.

Mammary duct ectasia emerged as the most plausible underlying pathology in the first case and remains the most frequently reported structural abnormality in children presenting with bloody nipple discharge. Unlike adult duct ectasia, which may be associated with chronic inflammation and nipple retraction, pediatric duct ectasia appears to reflect a transient, developmental ductal process with limited clinical consequences. Histopathological findings reported in surgically managed cases have consistently demonstrated benign ductal dilatation with periductal inflammation or fibrosis, without dysplasia or malignant change [1, 5]. Importantly, no cases of breast carcinoma have been reported in prepubertal children presenting with bloody nipple discharge, underscoring the benign nature of this entity.

Ultrasonography played a central role in the evaluation and management of the present cases. Breast ultrasound is widely regarded as the imaging modality of choice in pediatric patients, given its safety profile, lack of ionizing radiation, and ability to reliably demonstrate ductal dilatation and cystic changes. In the first case, ultrasound findings of a small retroareolar hypoechoic lesion with cystic ductal changes and absent vascularity were diagnostic of mammary duct ectasia and allowed confident exclusion of suspicious pathology. These characteristic sonographic features have been consistently reported and serve as a cornerstone for both diagnosis and parental reassurance [4, 6, 8, 9]. In the second case, normal ultrasonographic findings further supported a conservative approach, highlighting that structural abnormalities may not always be detectable despite the presence of clinical symptoms.

The management strategy adopted in both cases reflects the evolving consensus favoring expectant management. Earlier reports frequently described surgical excision or duct exploration, largely driven by diagnostic uncertainty and fear of malignancy. However, accumulating evidence demonstrates that surgical intervention does not alter clinical outcomes and carries the potential risk of damaging developing breast tissue, leading to future cosmetic or functional sequelae [1, 4, 5]. Contemporary practice emphasizes observation, reassurance, and serial clinical or sonographic monitoring, with spontaneous resolution typically occurring within several months. The time to resolution observed in the present cases falls well within the reported range and further supports this conservative paradigm.

Parental anxiety is a defining feature of pediatric bloody nipple discharge and represents a critical aspect of management. In the present series, detailed counseling and transparent discussion of the benign nature of the condition played a pivotal role in avoiding unnecessary investigations, particularly in the second case. This experience underscores the importance of physician familiarity with the condition and effective communication with caregivers. The inclusion of imaging findings in counseling discussions has been shown to enhance parental confidence and acceptance of non-interventional management [3, 4, 6].

The present report adds to the existing body of literature by reinforcing several key clinical principles. First, bloody nipple discharge in children should be approached as a distinct clinical entity from its adult counterpart. Second, ultrasound evaluation is sufficient in the absence of concerning clinical features. Third, conservative management with observation and parental reassurance is both safe and effective. Finally, awareness of this condition among primary care physicians, pediatricians, and surgeons is essential to prevent overtreatment.
